# Laparoscopic deroofing for a giant hepatic cyst with biliary communication: a case report

**DOI:** 10.1093/jscr/rjae176

**Published:** 2024-03-26

**Authors:** Taro Ikeda, Taro Okazaki, Yu Manabe, Rintaro Nakanishi, Hiroki Kagiyama, Yoshiyuki Owada, Masayoshi Hosono, Hiroyoshi Sendo

**Affiliations:** Department of Gastroenterological Surgery, Takatsuki General Hospital, Osaka 569-1192, Japan; Department of Gastroenterological Surgery, Takatsuki General Hospital, Osaka 569-1192, Japan; Department of Gastroenterological Surgery, Hyogo Cancer Center, Akashi, Hyogo 673-8558, Japan; Department of Gastroenterological Surgery, Takatsuki General Hospital, Osaka 569-1192, Japan; Department of Gastroenterological Surgery, Takatsuki General Hospital, Osaka 569-1192, Japan; Department of Gastroenterological Surgery, Takatsuki General Hospital, Osaka 569-1192, Japan; Department of Gastroenterological Surgery, Takatsuki General Hospital, Osaka 569-1192, Japan; Department of Gastroenterological Surgery, Takatsuki General Hospital, Osaka 569-1192, Japan; Department of Gastroenterological Surgery, Takatsuki General Hospital, Osaka 569-1192, Japan

**Keywords:** biliary communication, laparoscopic deroofing, simple hepatic cyst

## Abstract

Previous reports describing laparoscopic deroofing as a management modality for a hepatic cyst with biliary communication remain limited. We present the case of a 76-year-old woman who was monitored for 4 years for a giant hepatic cyst in the right lobe of the liver. She presented to our department with a chief complaint of abdominal distension. Moreover, imaging revealed a 24-cm giant hepatic cyst. During laparoscopic deroofing, minimal bile leakage from the intra-cyst wall was observed, which was laparoscopically closed with sutures. No bile leakage or cyst recurrence was observed 18 months postoperative. This highlights that laparoscopic surgery may be used in managing hepatic cysts with biliary communication. Intraoperative findings may reveal biliary communication, which requires careful observation of the cyst wall after deroofing.

## Introduction

Laparoscopic deroofing is a standard treatment for simple hepatic cysts [1]. Previous reports have described cases of deroofing for hepatic cysts with biliary communications, managed through open or laparoscopic surgery [2–9]. However, the optimal procedure for a hepatic cyst with biliary communication remains unestablished. Furthermore, the preoperative diagnosis of biliary communication may be difficult, and most cases are incidentally detected during surgery. We present a case of a giant hepatic cyst with biliary communication that was successfully managed through laparoscopic deroofing. Successful closure of the leakage site, to prevent postoperative bile leak, was achieved through laparoscopic suturing. Our case represents the largest instance among previously reported cases of cysts with biliary communication that were managed laparoscopically.

## Case report

**Figure 1:**
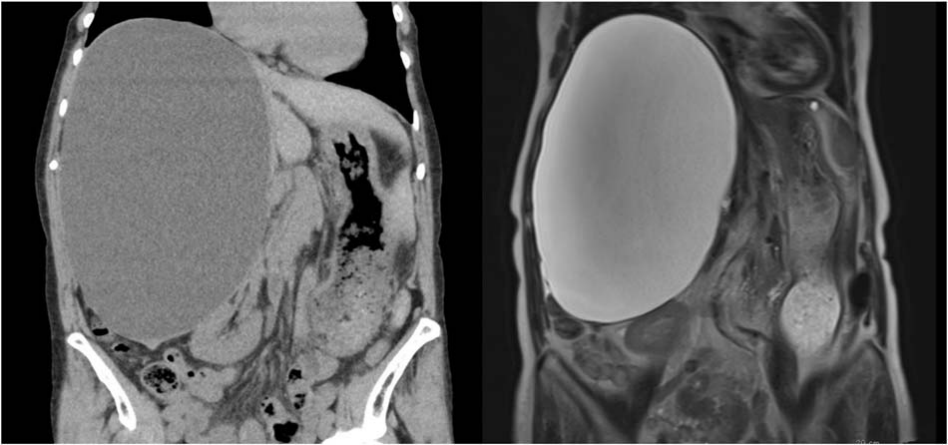
A 13 × 17 × 24 cm hepatic cyst in the right lobe is observed in both CT and MRI.

A 76-year-old woman, with an asymptomatic simple hepatic cyst in the right lobe of the liver, was closely monitored via magnetic resonance imaging (MRI) for 4 years. Only a slow annual increase in size was observed on MRI. One month before her surgery, the patient presented with abdominal distention. Blood examination for tumor markers, such as carcinoembryonic antigen and carbohydrate antigen 19-9, was within normal limits. Her biliary enzymes were slightly elevated (alkaline phosphatase of 151 U/l and γ-glutamyl trans peptidase of 61 U/l). A 13 × 17 × 24 cm hepatic cyst was observed in both computed tomography (CT) and MRI. Moreover, *T*_2_-weighted MRI revealed diffused high-signal intensity, without any tumor-like lesions such as filling defects or nodules (Fig. 1). Therefore, the diagnosis of a simple hepatic cyst was made, and she was recommended to undergo a laparoscopic deroofing for her symptomatic state.

**Figure 2:**
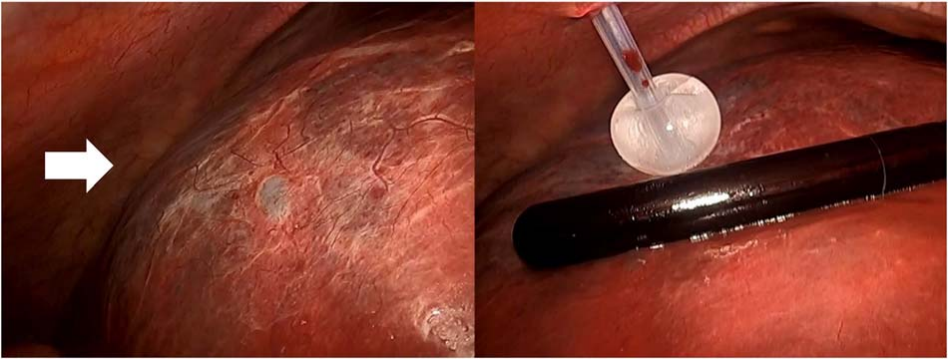
The hepatic cyst in the right lobe (arrow) and the puncture with a double-balloon catheter to aspirate the cystic fluid.

**Figure 3:**
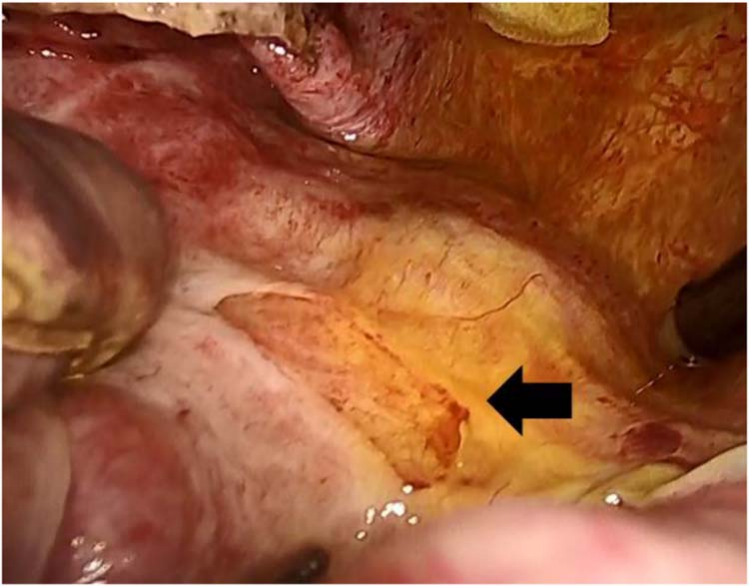
The bile leaked from the inner area of the cyst after deroofing (arrow).

**Figure 4:**
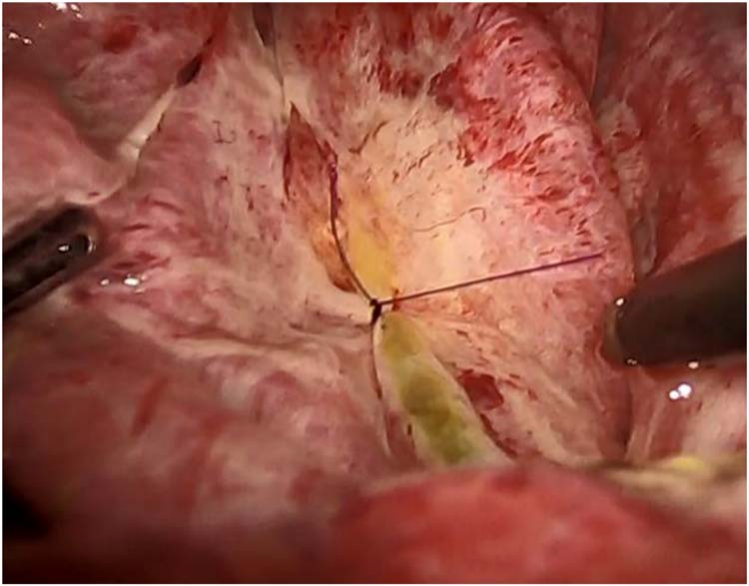
The orifice is closed laparoscopically with a monofilament absorbable suture.

The surgery required the deployment of four trocars. A 12-mm trocar was inserted through the umbilicus to establish pneumoperitoneum. Another 12-mm trocar was inserted below the xiphoid process, and 5-mm trocars were inserted in the right subcostal and flank regions. The boundary between the liver parenchyma and the cyst was determined via laparoscopic ultrasound sonography. A double-balloon catheter (S.A.N.D. balloon catheter, Hakko, Japan) was used to puncture the cystic wall and perform cystic fluid aspiration (Fig. 2). Cytological examination confirmed the absence of malignancy before deroofing. The cystic fluid was serous, with bilirubin levels within normal limits (total bilirubin of 0.1 mg/dl). Using a vessel sealing device (Ligasure Medtronic, USA), the cystic wall was deroofed, revealing a bile leak originating from the cyst’s inner region (Fig. 3). Laparoscopically, the orifice of the bile leakage was identified and closed using a 4-0 monofilament absorbable suture (Fig. 4 and Video 1). The cessation of the bile leakage was confirmed and a drainage tube was inserted. The total operative time was 193 minutes and the volume of fluid drained from the cyst was 3190 ml. No bile leakage was observed postoperatively, and she was discharged on the 7th postoperative day. Pathological examination of the excised cystic wall revealed a simple hepatic cyst. No cyst recurrence was observed via CT and ultrasound 18 months after surgery.

## Discussion

Despite the feasibility of laparoscopic deroofing, the use of the procedure in the management of a simple hepatic cyst with biliary communication remains unestablished. In our review of previous literature using the terms ‘liver cyst’ or ‘hepatic cyst’ and ‘biliary communication’ on PubMed, we identified eight studies where surgery was performed for hepatic cysts with biliary communication (Table 1) [2–9].

**Table 1:** Reported cases of a hepatic cyst with biliary communication managed surgically.

Author	Year	Age	Sex	Major axis (cm)	Location	Preoperative diagnosis of biliary communication	Open or laparoscopy	Treatment	Identification of the orifice
Ibrarullah MD [5]	1999	60	M	ND	Right lobe	No	Open	Deroofing + cystojejunostomy	Impossible
Masatsugu T [2]	2003	71	F	16	Right lobe	No	Laparoscopy	Deroofing + simple closure	Possible (intra-operative finding, cholangiography, and leak test)
Yamada T [6]	2009	56	F	10	Left lobe	Yes (cystography)	Laparoscopy	Deroofing	Impossible
Jain SK [7]	2010	17	F	16.3	Right lobe	Yes (ERC)	Open	Deroofing + cystojejunostomy	Impossible
Cui W [4]	2013	70	F	27.9	Right lobe	No	Open	Deroofing + simple closure	Possible (cholangiography)
Shimizu A [8]	2015	74	M	16	Right lobe	Yes (ERC)	Open	Deroofing + simple closure	Possible (leak test)
Shimada S [3]	2016	61	F	ND	Right lobe	Yes (DIC-CT)	Laparoscopy	Deroofing + simple closure	Possible (leak test)
Uylas U [9]	2019	23	M	ND	Left lobe	No	Open	Deroofing + simple closure	Possible (leak test)
Our case	2023	76	F	24	Right lobe	No	Laparoscopy	Deroofing + simple closure	Possible (intra-operative finding)

Abbreviation: ND, not described.

Cui *et al.* [4] performed deroofing for a 27.9-cm hepatic cyst through an open surgical approach. They argued that the closure of the orifice under laparotomy is a reasoned approach, and applicable to most cases. As summarized in Table 1, three other studies used laparoscopic surgery. Masatsugu *et al.* [2] and Shimada *et al.* [3] reported cases of hepatic cysts with biliary communication, managed with laparoscopic deroofing. They used laparoscopic suturing devices or intracorporeal ligation to close the orifice of the bile leakage. We were able to visualize the leakage site and successfully close the orifice in the giant cystic wall laparoscopically. Hence, we believe that the magnifying effect of laparoscopy is advantageous in confirming the site of the biliary communication and allows closure without postoperative bile leakage.

Making a preoperative diagnosis of biliary communication is challenging. According to Table 1, four cases were preoperatively diagnosed with biliary communication via endoscopic retrograde cholangiography (ERC), drip infusion cholecystocholangiography (DIC)-CT, or cystography. Additionally, one case had a ruptured cyst [3], and three cases underwent cystic fluid aspiration before surgery [6–8]. On the other hand, Masatsugu *et al.* reported that preoperative ERC failed to detect biliary communication, highlighting the difficulty of making a preoperative diagnosis. In our case, it was not until cystic fluid aspiration that the bile leakage was detected. The cystic fluid bilirubin level of our patient remained within normal limits. Previous literature and our case indicate that cystic fluid aspiration and the subsequent decrease in intra-cystic pressure led to a bile leakage from the cyst’s orifice. In such cases, preoperative DIC–CT or ERC might be unable to identify biliary communication, and incidental detection of biliary communication during surgery is likely. Therefore, careful observation of the intra-cystic wall after deroofing is recommended.

The reliable identification and closure of the bile leakage orifice are crucial to avoid postoperative bile leakage. However, some cases are present where detecting the orifice intraoperatively may be difficult. Studies have described the efficacy of performing an intraoperative leak test [3, 8, 9]. Shimada *et al.* [3] succeeded in finding the orifice by performing a leak test with indigo carmine under laparoscopy. This highlights that performing an intraoperative leak test is recommended in cases where the leakage site is not easily visible. Moreover, Hanaki *et al*. [10] reported that fluorescence imaging using indocyanine green (ICG) via intravenous injection was effective for detecting a bile leakage during hepatectomy including laparoscopic resection. Fluorescence imaging by systemically administered ICG may be a minimally invasive procedure and applicable for the management of incidentally detected a bile leakage during laparoscopic deroofing.

In conclusion, we believe that laparoscopic surgery holds promise as a valuable and safe approach to managing giant hepatic cysts with biliary communication, allowing identification and closure of the bile leakage orifice.

## Supplementary Material

video_rjae176 The orifice of the bile leakage was identified and closed laparoscopically with a monofilament absorbable suture.
